# Nocturia Frequency and Its Association with Sleep Disturbance and Health-Related Quality of Life in a Urology Outpatient Population

**DOI:** 10.3390/jcm15072492

**Published:** 2026-03-24

**Authors:** Theodore Voudoukis, Francesk Mulita, Vasileios Leivaditis, Ejona Shaska, Andreas Antzoulas, Dimitrios Litsas, Panagiotis Dimitrios Papadopoulos, Elias Liolis, Konstantinos Tasios, Paraskevi Katsakiori, George Theofanis, Ioannis Maroulis, Georgios Tsakaldimis

**Affiliations:** 1 Department of Urology, General Hospital of Eastern Achaia—Unit of Aigio, 25100 Aigio, Greece; thvoudoukis@gmail.com; 2 Department of Surgery, General University Hospital of Patras, 26504 Patras, Greece; a.antzoulas@hotmail.com (A.A.); kostastasiosmd@gmail.com (K.T.); vkatsak@gmail.com (P.K.); gtheofanis3@yahoo.gr (G.T.); 3 Department of Cardiothoracic and Vascular Surgery, Westpfalz Klinikum, 67655 Kaiserslautern, Germany; vnleivaditis@gmail.com; 4 Department of Emergency Psychiatry, “Ali Mihali” Psychiatric Hospital, 9401 Vlora, Albania; zilja.jona@yahoo.it; 5 Department of Surgery, General Hospital of Lamia, 35100 Lamia, Greece; dimlitsas@icloud.com; 6 Department of General Surgery, Spital Herisau, 9100 Herisau, Switzerland; panospapado1997@icloud.com; 7 Department of Oncology, General University Hospital of Patras, 26504 Patras, Greece; lioliselias@yahoo.gr; 8 Department of Urology, General University Hospital of Alexandroupolis, Democritus University of Thrace, 68100 Alexandroupolis, Greece; g.tsakaldimis@hotmail.com

**Keywords:** nocturia, quality of life, mental health, sleep disorders

## Abstract

**Objective**: Nocturia, defined as waking from sleep to void, is a frequent lower urinary tract symptom associated with impaired sleep quality and reduced quality of life. This study aimed to evaluate the prevalence of nocturia episodes and their impact on sleep disturbance and health-related quality of life. **Methods**: A questionnaire-based cross-sectional study was conducted at the Urology Outpatient Clinic of the General Hospital of Eastern Achaia between November 2023 and May 2024. Participants reporting nocturia were assessed using the Nocturia Quality of Life (N-QOL) questionnaire, the Athens Insomnia Scale (AIS), and the EQ-5D questionnaire. Demographic data and comorbid conditions were also collected. Univariate analyses and multiple linear regression were applied to identify factors associated with nocturia-related outcomes. **Results**: A total of 89 participants (78 men and 11 women; mean age 68.9 years) were included. Most participants reported 2–3 nocturnal voids per night. The N-QOL score was significantly associated with the frequency of nocturia episodes (r = −0.55, *p* < 0.0001), and regression analysis confirmed this relationship (coefficient: −6.7; 95% CI: −10.4 to −3.1). Individuals scoring ≥ 8 on the OAB-V8 scale demonstrated significantly lower N-QOL performance. **Conclusions**: Increasing nocturia frequency is associated with impaired sleep, reduced vitality, and diminished quality of life, particularly among older adults. Nocturia should be recognized as a clinically relevant symptom requiring targeted evaluation and personalized management strategies.

## 1. Introduction

Nocturia is a prevalent and clinically relevant lower urinary tract symptom (LUTS) defined by the International Continence Society (ICS) as the need to wake from sleep one or more times to void [1]. The terminology and clinical definition used in this manuscript follow ICS standards, specifying that nocturnal voiding occurs after sleep onset and before final awakening. While a single nighttime void may be tolerated by many individuals, recurrent awakenings are associated with impaired daily functioning, reduced vitality, and diminished well-being [1,2]. Nocturia is therefore recognized as a symptom reflecting underlying physiological or pathological processes rather than a normal consequence of aging.

According to the International Classification of Diseases (ICD-11), nocturia is classified as a lower urinary tract symptom and defined as waking one or more times during the main sleep period to void (ICD-11: MB40.0). This definition aligns with ICS terminology, distinguishing nocturia as a clinical symptom linked to sleep interruption and urinary physiology rather than a behavioral or psychiatric condition. Importantly, nocturia is not defined in the DSM-5, as it does not constitute a mental health disorder; instead, it may contribute secondarily to psychological distress or reduced well-being through sleep fragmentation and chronic symptom burden.

Nocturia is highly prevalent in clinical practice, particularly among older adults, where it is frequently associated with fragmented sleep, daytime fatigue, reduced productivity and diminished quality of life [3,4]. Despite this clinical relevance, evidence regarding nocturia and its impact on sleep and health-related quality of life in Greek clinical settings remains limited. Understanding patient-reported outcomes is essential, as nocturia represents a subjective and multifactorial symptom that cannot be fully characterized by urological parameters alone. The Athens Insomnia Scale (AIS) provides a standardized measure of sleep disturbance, and the EQ-5D assesses health-related quality of life, including a single dimension on anxiety/depression [5,6,7]. These tools do not assess mental health as a clinical construct; rather, they offer insight into how nocturnal awakenings influence sleep quality and perceived well-being.

Given the increasing recognition of nocturia as a complex symptom associated with reduced life satisfaction and impaired functioning, further research is warranted to better characterize its burden in real-world clinical populations. The primary aim of this study was to examine the frequency of nocturia episodes and their association with sleep disturbance and health-related quality of life in individuals presenting to a urology outpatient clinic. By focusing on patient-reported outcomes, this study seeks to contribute to a more comprehensive understanding of nocturia and its implications for daily living. Given the limited research conducted in Greek clinical populations, the study focuses on individuals presenting to a urology outpatient clinic, without aiming to establish population-level prevalence.

## 2. Nocturia

Nocturia is not a symptom exclusive to older adults, as it may occur at any age. Its estimated prevalence in children is approximately 15% at the age of five, decreasing to about 4% in adolescence [5]. The prevalence then increases substantially in adulthood, reaching 58% and 66% among men and women aged 50–59 years, respectively, and rising further to 72% and 91% in individuals over 80 years of age [6,7].

This age-related increase is not only attributable to the burden of comorbid conditions frequently observed in older individuals but also to physiological changes associated with aging. The kidneys progressively lose their ability to concentrate urine, which leads to increased nocturnal urine production when renal perfusion rises in the recumbent position during sleep [8,9]. Patients with nocturia may experience varying degrees of distress: some are significantly bothered by their symptoms, while others consider the condition tolerable. Ultimately, the level of discomfort often determines whether individuals seek medical attention [1].

**Figure 1:**
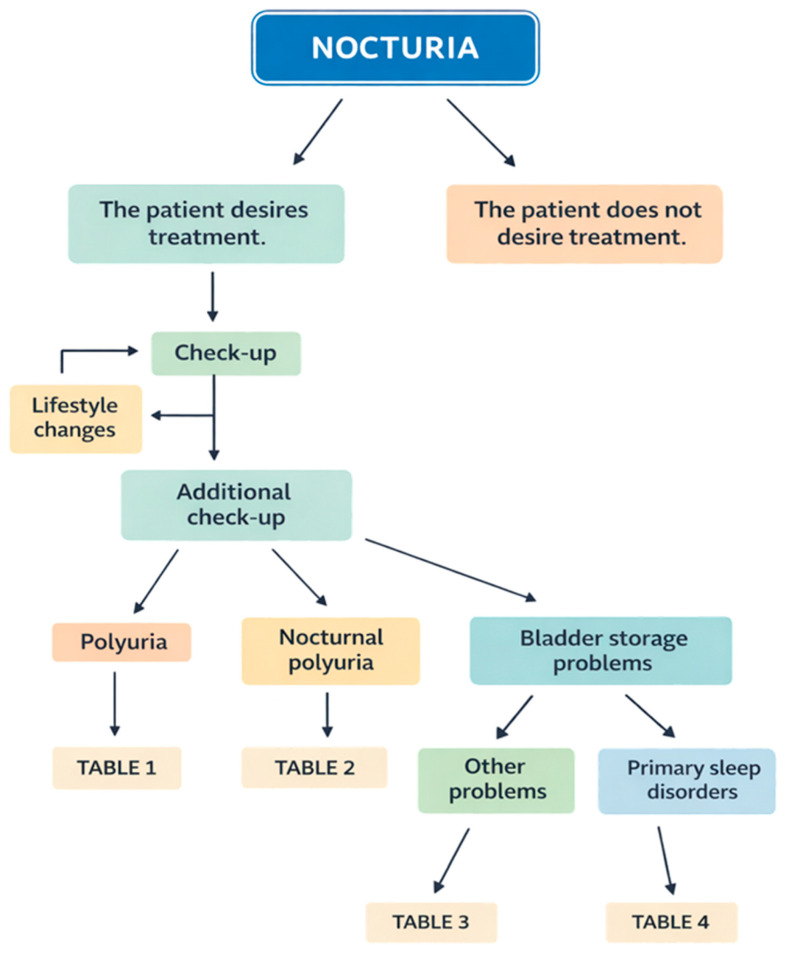
A simple schematic assessment of nocturia.

The initial assessment of nocturia is guided by a simple diagnostic algorithm, illustrated schematically in Figure 1.

Like urinary frequency, nocturia may result from either increased urine production or reduced bladder storage capacity [1]. Daytime urinary frequency in the absence of nocturia is often psychogenic in origin and is most commonly associated with anxiety disorders [10]. Nocturia can also occur in individuals who consume large volumes of fluid late in the day, particularly caffeinated or alcoholic beverages, which exert a pronounced diuretic effect [11,12].

Patients presenting with nocturia may seek consultation from various medical specialties, each applying its own clinical approach. A standardized definition is therefore essential to ensure consistent diagnosis and therapeutic decision-making across disciplines [13,14]. Shared clinical terminology promotes uniform evaluation and management and reduces fragmentation in care.

Lifestyle modification represents the first-line intervention. Reducing the intake of caffeine, alcohol, and fluids during the evening hours may be beneficial. Extreme fluid restriction, however, should be avoided, particularly in individuals with unrecognized diabetes insipidus [12]. If these initial measures fail to improve symptoms, patients should be advised to seek further clinical evaluation [13,14].

Diagnostic workup should begin with a 1–3-day voiding diary documenting fluid consumption, timing and volume of urination, sleep patterns, and subjective sleep quality. Although assessing quality of life is crucial, no single questionnaire comprehensively captures the overall burden of nocturia [15,16]. In cases where nocturia coexists with suspected sleep pathology, polysomnography (PSG) may aid in identifying contributory disorders [17,18]. A thorough and systematic evaluation enables identification of the underlying etiology and supports individualized treatment planning [15,16].

### 2.1. Nocturia, Sleep Disorders & Quality of Life

In older adults, particularly those with insomnia, frequent nocturnal voiding further disrupts already compromised sleep quality. Numerous studies have demonstrated that increasing nighttime urination is associated with poorer sleep outcomes [19]. Nocturia may cause sleep disturbances in up to 75% of elderly individuals [6]. More than 60% of older adults experience nocturia, although the condition remains largely underrecognized by the general population. The resulting sleep loss and fragmentation can lead to fatigue, mood changes, daytime somnolence, reduced productivity, impaired concentration, elevated accident risk, and cognitive decline [20,21]. Notably, approximately 25% of falls among older individuals occur at night, with a substantial proportion of these events happening when patients rise to void [1]. Furthermore, nocturia has been associated with increased morbidity and mortality [22,23,24].

Nocturia negatively affects not only sleep, but also multiple dimensions of quality of life, often extending to the patient’s partner or family [25,26]. Both the frequency of nocturnal episodes and the timing of awakenings play a critical role in determining disease burden. Given these wide-ranging consequences, nocturia must be recognized as an important health condition that contributes to substantial morbidity, reduced life satisfaction, and psychological and social strain. Diagnostic tools are available to facilitate early and accurate identification, and several therapeutic approaches exist to alleviate symptoms [3,27]. Nevertheless, additional research is required to guide the development of targeted and individualized treatment strategies.

Although several instruments for evaluating quality of life are available, the concept of Quality-Adjusted Life Years (QALYs) has gained notable attention among clinicians and health economists over the past two decades. QALYs integrate life expectancy with quality of life and represent the number of years lived in full health [28,29]. This internationally recognized metric, introduced in the 1970s, is calculated by weighting the duration spent in a given health state with a utility score derived from standardized assessments [30]. Despite being less sensitive to chronic conditions and preventive interventions, QALYs may offer valuable insights for assessing treatment strategies and healthcare interventions for nocturia [31].

### 2.2. Causes of Nocturia

#### 2.2.1. Polyuria

Polyuria is defined as a 24-h urine output exceeding 40 mL per kilogram of body weight—equivalent to more than 3000 mL per day in an adult weighing approximately 70 kg [32,33]. When polyuria is suspected, diagnostic evaluation should first determine whether the condition is attributable to diabetes mellitus or diabetes insipidus, and subsequently identify the specific subtype (Table 1).

Assessment typically includes measurement of glucose levels in serum and urine over a 24-h period, complemented by targeted diagnostic tests to differentiate among etiologies and resolve the diagnostic uncertainty [34,35].

#### 2.2.2. Nocturnal Polyuria

Nocturnal polyuria refers to the excessive production of urine during nighttime sleep. Measurement includes the total urine volume produced from the time an individual lies down to sleep until the first awakening to void [11]. In younger individuals, nocturnal polyuria is defined as nighttime urine output exceeding 20% of the total 24-h urine volume, whereas in individuals aged 65 years or older, this threshold increases to more than 33% [11,36]. In such cases, the increased nocturnal urine production is typically accompanied by a compensatory reduction in daytime output, resulting in a preserved total 24-h urine volume. The principal etiologies of nocturia are summarized in Table 2 [10].

Certain populations represent exceptions to these criteria. Patients with diabetes insipidus and individuals whose sleep duration significantly deviates from the standard eight-hour cycle may demonstrate altered urine output patterns that fall outside conventional definitions [3]. Nocturnal polyuria is also common among patients with congestive heart failure or peripheral edema, in whom recumbency increases venous return and intravascular volume, subsequently enhancing renal perfusion and urine production during sleep [8].

#### 2.2.3. Bladder Storage Disorders

Patients with nocturia who do not exhibit polyuria are more likely to experience reduced bladder storage capacity or disturbances in sleep architecture. Reduced functional bladder capacity can be identified through the use of a voiding diary, comparing the nocturnal voided volume with the individual’s maximum functional bladder capacity at any given time [37]. However, the distinction between normal and abnormal nocturnal urine volumes is not clearly standardized, and relying solely on this comparison may lead to inaccurate assessment (Table 3). To more accurately evaluate nocturia, several quantitative indices have been developed, including the nocturia index—defined as the ratio of average nocturnal urine volume to functional bladder capacity—and the nocturnal polyuria index, calculated as the ratio of average nocturnal urine volume to total 24-h urine output [38].

This table highlights several factors that impair bladder storage capacity and can contribute to symptoms such as urgency, incontinence, or nocturia [39]. In men, benign prostatic hyperplasia is the most prevalent cause of obstructive lower urinary tract symptoms, including nocturia [40,41]. In women, nocturia is most commonly associated with overactive bladder, a condition that frequently remains undiagnosed [42].

It is also important to recognize that, despite suggestive voiding diaries, the underlying cause of nocturia may be a sleep disorder. Individuals who awaken during the night and subsequently void—even when passing only small volumes of urine—may suffer from sleep-related disturbances [19,43]. Additional sleep disorders associated with nocturia are listed in Table 4 [19,43]. In such cases, accurate diagnostic evaluation is critical to ensure appropriate management.

### 2.3. Treatment of Nocturia

The primary objective in treating nocturia is symptom reduction. Therefore, identifying the underlying etiology—whether behavioral, medical, or surgical—is essential in guiding appropriate management [1]. The effectiveness of treatment varies according to contributing factors and is closely linked to improvements in patient well-being. Core therapeutic goals include decreasing the number of nocturnal voids, minimizing symptom-related distress, prolonging the time before the first awakening, increasing total sleep duration, and addressing comorbid medical conditions [1,4].

Initial management typically focuses on lifestyle modification, although evidence from controlled clinical trials remains limited. Fluid intake during the evening and caffeine consumption in older adults do not appear to be directly associated with increased nocturia, and a 25% reduction in overall daily fluid intake has demonstrated limited clinical benefit [44]. Physical exercise, particularly pelvic floor muscle training and bladder retraining, has proven effective for patients with overactive bladder [45]. Furthermore, engaging in a 30-min evening walk over an eight-week period has been associated with a reduction in nocturia episodes and a 50% improvement in quality-of-life scores [46]. These findings underscore the role of physical activity in enhancing bladder function and patient well-being, although many individuals struggle to modify established lifestyle habits.

Pharmacological therapy is typically considered when behavioral measures are insufficient. Desmopressin, a synthetic analogue of the antidiuretic hormone, is widely used in both pediatric and adult populations. Administered orally or intranasally, it reduces urine production for approximately 7–12 h and maintains therapeutic efficacy for more than 12 months [47]. Notably, desmopressin has been shown to reduce nocturia episodes by more than 50%, with a 34% reduction in nocturnal urine volume observed in women and a 20% reduction in men [48].

Loop diuretics, administered 4–6 h before bedtime, represent an alternative or adjunctive strategy, provided that the patient does not present with obstructive uropathy [49]. Among these, furosemide is the most potent short-acting agent. Combination therapy with furosemide and desmopressin has demonstrated significant reductions in nocturnal urine output and nocturia frequency, while also improving the duration of uninterrupted sleep [50]. These interventions have been associated with improved sleep continuity, enhanced quality of life, and increased productivity.

Antimuscarinic agents are frequently used in patients with nocturia secondary to detrusor overactivity. While clinical outcomes vary, nighttime administration can reduce urgency-associated nocturnal voiding [51]. α-blockers have been shown to alleviate nocturia and lower urinary tract symptoms in patients with benign prostatic hyperplasia, while also improving the nocturnal polyuria index and restoring circadian rhythms of urine production [52].

Surgical interventions for benign prostatic hyperplasia may lead to short-term reductions in nocturia; however, among lower urinary tract symptoms, nocturia tends to show minimal sustained improvement over time [40].

Sedatives and hypnotics have demonstrated efficacy in the short-term management of nocturia and may also benefit male patients with benign prostatic hyperplasia who have an inadequate response to α-blocker therapy [53]. Additionally, treatment of obstructive sleep apnea with continuous positive airway pressure (CPAP) has been shown to reduce nocturia episodes in older adults [54].

Ultimately, the effectiveness of nocturia treatment should be assessed from a patient-centered perspective, taking into account the degree of symptom-related discomfort and its impact on daily functioning. Further research is needed to better characterize the economic burden of nocturia, elucidate its pathophysiological mechanisms, and clarify its interactions with sleep stages and sleep disorders.

## 3. Materials and Methods

### 3.1. Study Setting and Participants

This study employed an observational, cross-sectional design conducted at the Urology Outpatient Clinic of the General Hospital of Eastern Achaia. The primary variable was the frequency of nocturnal awakenings to void. Secondary variables included sleep disturbance measured by the Athens Insomnia Scale (AIS), nocturia-related quality of life measured by the Nocturia Quality of Life questionnaire (N-QOL), and health-related quality of life measured using the EQ-5D and EQ-VAS instruments (EuroQol Group, Rotterdam, The Netherlands). Demographic and clinical characteristics (age, gender, diabetes mellitus, benign prostatic hyperplasia, overactive bladder symptoms, and use of diuretics) were evaluated as potential explanatory or modifying factors (Supplementary Materials).

All patients presenting to the clinic between November 2023 and May 2024 who reported nocturia were invited to participate. The clinic serves a mixed population from both urban and rural regions of Aigialeia and Kalavryta. Participants were recruited using consecutive non-probability sampling, including all individuals who reported nocturia during their visit to the Urology Outpatient Clinic within the study period.

This study was designed as a clinic-based observational analysis aiming to evaluate associations between nocturia frequency and patient-reported outcomes within a real-world urological setting. It was not intended to estimate population-level prevalence. Consecutive sampling was applied to minimize selection bias within the outpatient population and to reflect routine clinical practice.

### 3.2. Inclusion and Exclusion Criteria

Inclusion criteria were:

- Self-reported nocturia (≥1 nocturnal voiding episode),
- Age ≥ 18 years,
- Ability to understand and complete the questionnaire.

No exclusion criteria were applied, as the aim was to capture real-world presentations of nocturia. All individuals who reported nocturia were eligible for inclusion, irrespective of additional urological or non-urological conditions, psychological status, ongoing life stressors, or concurrent medications. This approach was adopted to preserve ecological validity and accurately reflect the clinical population presenting with nocturia in this healthcare setting.

### 3.3. Data Collection Procedure

After obtaining informed consent, participants completed a structured questionnaire administered in paper form during the clinic visit. Demographic characteristics (age, gender, marital status, employment, and education), medical history (diabetes mellitus, benign prostatic hyperplasia, overactive bladder), current medication use (including diuretics, anxiolytics, and antidepressants), and daily fluid intake were recorded. Pairwise post hoc comparisons were not conducted, as the analyses aimed to assess overall group effects rather than identify specific pairwise differences.

### 3.4. Assessment Instruments

#### 3.4.1. N-QOL (Nocturia Quality of Life Questionnaire)

The Nocturia Quality of Life (N-QOL) questionnaire evaluates the impact of nocturia on daily functioning and psychological burden. It consists of 12 items scored on a 5-point Likert scale (0–4). The items are grouped into two domains:

- Sleep/Energy (questions 1–5 and 7),
- Bother/Concern (questions 6 and 8–12).

Scores are converted to a 0–100 scale, with higher scores indicating better quality of life. The Greek version of the questionnaire was approved by the Mapi Research Institute in 2006 to ensure conceptual equivalence.

#### 3.4.2. OAB-V8 (Overactive Bladder Awareness Tool)

The OAB-V8 consists of 8 items scored on a 0–5 scale (0 = “not at all” and 5 = “very much”). It screens for symptoms associated with overactive bladder, including urgency, urge incontinence, daytime frequency, and nocturia. A total score ≥ 8 indicates probable overactive bladder. The validated Greek version was certified in 2005 by Corporate Translations Inc.

#### 3.4.3. Athens Insomnia Scale (AIS)

The Athens Insomnia Scale (AIS) comprises 8 items assessing nocturnal sleep disturbance and daytime functioning. Each item is scored from 0–3 or 0–4, yielding a total score from 0–24. The first five items evaluate sleep initiation, awakenings, duration, and quality, while the last three measure fatigue, functioning, and well-being during the day. Higher scores indicate greater severity of insomnia. The AIS is validated in Greek and based on ICD-10 diagnostic criteria.

#### 3.4.4. EQ-5D

The EQ-5D assesses health-related quality of life across five domains: mobility, self-care, usual activities, pain/discomfort, and anxiety/depression. Each dimension is rated on a three-level scale (1 = no problem, 2 = moderate problems, 3 = severe problems), enabling 243 possible health states. Respondents also complete the EQ-VAS, a visual analogue scale rating global health from 0 (“worst imaginable health”) to 100 (“best imaginable health”). The Greek version was validated in 2001.

### 3.5. Ethical Considerations

The study was approved by the Scientific Committee of the General Hospital of Eastern Achaia. Participation was voluntary, and anonymity was ensured. All procedures complied with the principles of the Declaration of Helsinki.

### 3.6. Objective of the Study and Hypothesis

The objective of this study was to characterize nocturia in a cohort of patients presenting to the Urology Outpatient Clinic of the General Hospital of Eastern Achaia and to examine its association with sleep quality and health-related quality of life. Rather than estimating population-level prevalence, the study reports the incidence of nocturia among clinic attendees during the study period.

A secondary objective was to evaluate the relationship between the number of nocturnal voids and patient-reported outcomes, including sleep disturbance (AIS), nocturia-related quality of life (N-QOL), and perceived health status (EQ-5D/EQ-VAS). We further explored whether demographic or clinical factors (BPH, diabetes, OAB symptoms) predicted poorer outcomes.

We hypothesized that the frequency of nocturnal awakenings would be negatively associated with quality-of-life indicators and sleep quality. Specifically, we expected individuals with more nocturia episodes to report lower N-QOL scores, greater sleep disruption on the Athens Insomnia Scale, and reduced health-related well-being on EQ-5D/EQ-VAS. We further hypothesized that clinical factors such as benign prostatic hyperplasia or symptoms of overactive bladder would be associated with poorer outcomes.

## 4. Statistical Analysis

Responses from all subsections of the questionnaire were initially compiled. In accordance with the scoring protocol of the OAB instrument, an additional 2 points were added to the total score of each male participant.

Given the modest sample size and the risk of inflated type I error due to multiple testing, post-hoc pairwise comparisons were not systematically performed; the analysis focused primarily on overall group effects and multivariable modeling. Interaction terms were not included in multivariable models due to limited subgroup size and the risk of model overfitting.

For the N-QOL questionnaire, two subscales were constructed:

- Sleep/Energy, comprising six items related to concentration, energy, daytime sleepiness, productivity, instrumental daily activities, and sleep–wake disturbances.
- Bother/Concern, comprising six items addressing concerns about water intake, disturbing others at home, awakening due to urinary urge, fear of symptom progression, apprehension regarding ineffective treatment, and general nocturia-related distress.

The following score transformations were applied:

- N-QOL total score: 0–100
- Sleep/Energy and Bother/Concern subscales: 0–50 each
- Athens Insomnia Scale: 0–100
- EQ-5D: 0–10

For all scales, lower values indicated worse outcomes, while higher values reflected more favorable conditions.

Continuous variables were expressed as mean ± standard deviation. Univariate analyses were performed to examine relationships between questionnaire scores and demographic or clinical variables. The Mann–Whitney U test was used to compare two independent groups, whereas the Kruskal–Wallis test was applied for comparisons across more than two groups. Non-parametric tests were chosen instead of *t*-tests or ANOVA due to significant skewness in the distribution of scores and the relatively small sample size.

These non-parametric methods are not inferior in efficiency when normality assumptions are violated and show only limited loss of efficiency when the distribution is normal. Categorical variables, expressed as percentages, were compared using the Mantel–Haenszel chi-square test for linear trend or Pearson’s chi-square (χ^2^). Fisher’s exact test was employed when expected cell counts were fewer than five. Spearman rank correlation coefficients were calculated to assess associations between quantitative variables.

Distributional assumptions were examined and the scale variables demonstrated marked skewness. Given the non-normality of the data and the limited sample size, non-parametric methods were applied throughout the analysis. Independent group comparisons were performed using the Mann–Whitney U or Kruskal–Wallis test, while correlations between quantitative variables were evaluated using Spearman rank coefficients.

Univariate analyses were used to explore the effect of individual parameters, followed by multivariable regression models to evaluate the independent influence of these variables while controlling for potential confounders. Statistical significance was set at *p* < 0.05. All analyses were performed using SAS statistical software, version 9.3.

## 5. Results

The study was conducted between November 2023 and May 2024 and included 89 participants with a mean age of 68.6 years. All individuals attended the regular morning Urology Outpatient Clinic of the General Hospital of Eastern Achaia, which operates twice weekly. Participants were enrolled if nocturia was either their primary complaint or part of a broader clinical presentation. Given the exploratory nature of this study and the limited literature on nocturia in Greek clinical populations, item-level distributions from all validated instruments are reported to provide greater transparency and allow a detailed interpretation of patient-reported symptom burden and functional impact.

A statistically significant age difference was observed between male and female participants (*p* < 0.0001). The mean age of men was 70.6 ± 9.21 years, whereas that of women was 54.1 ± 15.6 years. Among women, 63.6% were younger than 60 years, while 76.9% of men were between 60 and 79 years of age. Additionally, 15.7% of participants were older than 80 years, almost exclusively men (Table 5).

Most individuals were married and had two children (47.19%). The majority were unemployed (75.3%), and 70.8% had primary-level education. These characteristics are largely attributable to the demographic composition of the clinic’s population, which predominantly consisted of retired male farmers.

Responses to the primary questionnaire item assessing nocturnal voiding frequency are presented in Table 6. The majority of participants reported a moderate degree of nocturia, corresponding to 2–3 nighttime awakenings. A considerable proportion (21.3%) experienced severe nocturia. It is noteworthy that, although a single nocturnal void is often considered clinically insignificant, 9% of respondents—comprising exclusively individuals younger than 64 years and employed—reported being bothered by waking once to urinate. This finding highlights that even low-frequency nocturia can be perceived as distressing and lead to healthcare-seeking behavior.

Analysis of the N-QOL questionnaire responses indicated that the most prominent issue associated with nocturia was the discomfort caused by waking during the night to void (1.78 ± 0.97). This was followed by concern regarding potential symptom progression (1.71 ± 1.19), which may be compounded by anxiety about the lack of effective treatment options (1.56 ± 1.12). A similar burden was observed for reduced nocturnal sleep (1.57 ± 1.20), concern about the need to wake to urinate (1.38 ± 1.31), perceived need for daytime rest (1.35 ± 1.12), and low energy levels the following day (1.26 ± 1.14).

Additional difficulties were reported to a lesser extent, including reduced productivity (1.17 ± 1.17), impaired concentration (1.15 ± 1.17), and diminished engagement in preferred activities (0.96 ± 1.11). Less frequent concerns involved the possibility of disturbing others at home (0.66 ± 1.01) and increased attention to fluid intake (0.53 ± 0.71). These findings are summarized in Table 7. While overall scores provide a general indication of quality of life and sleep disturbance, item-level distributions illustrate the specific domains in which nocturia affects patients, allowing clinicians to appreciate the heterogeneity of its impact.

Responses to the overactive bladder (OAB) items indicated that the greatest discomfort was associated with awakening during the night (2.55 ± 1.32) and nocturnal urination (2.53 ± 1.35), with approximately one in three participants reporting significant or very significant distress. These were followed by frequent daytime urination (1.74 ± 1.49). The remaining items reflected substantially lower levels of discomfort, with mean scores ranging from 0.70 to 0.47 (Table 8).

The sleep questionnaire revealed that the most prominent issue was awakening during the night (1.57 ± 0.66), with 98.9% of participants reporting at least a minor disturbance. This was followed by problems related to perceived sleep quality, total sleep duration, sleep onset, and final awakening relative to the desired time. Additional difficulties included reduced well-being, daytime drowsiness, and impaired functionality the following day (Table 9).

Finally, analysis of the health status questionnaire showed that the most frequently reported issue was anxiety/depression, affecting 61.8% of participants (0.80 ± 0.73). Substantially lower levels of concern were observed for other dimensions, including pain/discomfort (0.19 ± 0.42), mobility problems (0.13 ± 0.34), difficulties with daily activities (0.12 ± 0.33), and limitations in self-care (0.09 ± 0.29) (Table 10).

A total of 15 out of the 243 possible EQ-5D health states were reported. The most frequent were health states 11111 (full health) and 11112 (moderate anxiety/depression), each accounting for 33.7% of participants. These were followed by state 11113 (severe anxiety/depression), observed in 12.3% of respondents. For these three states, the mean EQ-VAS scores were similar (71.3, 71.4, and 72.7, respectively). Notably, the mean EQ-5D index score was high (0.826), indicating that the majority of participants perceived themselves to be in very good overall health (Table 11).

The potential factors that could influence quality of life and sleep among individuals reporting nocturia were examined using univariate analyses. These variables included the number of nocturia episodes, demographic characteristics (age, gender, marital status, employment status, and educational level), diabetes mellitus (DM), benign prostatic hyperplasia (BPH), the overactive bladder score, and daily fluid consumption (Table 12).

The use of the Overactive Bladder questionnaire (OAB-V8) in this study served two primary purposes. First, overactive bladder syndrome has been consistently shown to be strongly associated with nocturia, as well as with impaired sleep and reduced quality of life—even among individuals who remain unaware of their condition, a phenomenon that appears to be relatively common in the Greek population. In addition, OAB is frequently linked to conditions involved in the development and pathophysiology of nocturia, such as diabetes mellitus and benign prostatic hyperplasia.

Second, the instrument was included to facilitate a general estimation of how frequently symptoms suggestive of detrusor overactivity are present among individuals with nocturia. Given the potential influence of this factor on nocturia severity, a preliminary exploratory analysis was deemed necessary. Consequently, although a univariate analysis was performed, the results are presented descriptively (Table 12) without detailed interpretation, as further emphasis would extend beyond the scope of this study and would not meaningfully contribute to its primary research objectives.

The analysis demonstrated that an OAB-V8 score of ≥8 occurred more frequently with increasing age, rising from 50.0% in participants younger than 60 years to 85.7% in those aged 80 years or older (*p* = 0.040). A statistically significant difference was also observed with respect to educational level: individuals with basic or technical education scored ≥ 8 at rates of 66.7% and 82.3%, respectively, while only 11.1% of participants with higher education showed similarly elevated scores (*p* = 0.034).

Employment status also showed a statistically significant association, with 70.1% of employed individuals demonstrating OAB-V8 scores ≥ 8, compared with 45.5% of unemployed participants (*p* = 0.036). Although no statistically significant difference was identified with respect to gender alone, stratification by benign prostatic hyperplasia (BPH) status among men yielded meaningful distinctions (*p* = 0.001). Specifically, 87.5% of men with BPH had scores ≥ 8, compared with 45.3% of men without BPH and 72.7% of women. For the remaining parameters examined, no statistically significant associations were observed.

Univariate analysis revealed that nocturia severity had a significant impact on N-QOL scores. Mean values decreased progressively with increasing number of nocturnal awakenings, declining from 84.4 ± 13.4 among individuals who woke once to 43.8 ± 17.1 among those who woke five or more times (*p* < 0.0001). Similar trends were identified for the Sleep/Energy subscale (ranging from 43.0 ± 8.2 to 18.0 ± 12.2; *p* < 0.0001) and the Bother/Concern subscale (ranging from 41.4 ± 7.7 to 24.8 ± 6.8; *p* = 0.0003). Spearman correlation coefficients further supported these findings (N-QOL: r = −0.55, *p* < 0.0001; Sleep/Energy: r = −0.49, *p* < 0.0001; Bother/Concern: r = −0.57, *p* < 0.0001) (Table 13).

The remaining survey data revealed that only 9 participants (10.1%) were unmarried or divorced. Approximately 70% had completed basic education, nearly 20% had received technical or vocational training, and about 10% held a higher education degree. In addition, three out of four participants were not employed, predominantly due to retirement. Based on the OAB-V8 questionnaire, 64.0% of respondents scored ≥ 8, indicating a strong likelihood of overactive bladder syndrome. Among male participants, 32 of 78 (41.0%) reported benign prostatic hyperplasia. The influence of these parameters on quality of life in individuals with nocturia was assessed using the N-QOL total score and its subcomponents (Table 13).

No statistically significant differences were observed across the three N-QOL scales with respect to age at the 5% level, although the lowest values were recorded in individuals aged ≥ 80 years. Women showed lower scores than men on the Sleep/Energy subscale (26.3 ± 13.9 vs. 35.6 ± 12.7, *p* = 0.034), whereas no statistically significant differences were found between sexes for the remaining two subscales. When men were stratified according to the presence of BPH, statistically significant differences emerged across all three scales (*p* = 0.004 for N-QOL; *p* = 0.016 for Sleep/Energy; *p* = 0.005 for Bother/Concern). No statistically significant associations were observed with marital or employment status. Educational level, however, demonstrated a significant effect: participants with technical education had lower scores across all three scales compared with those with basic education, whereas individuals with higher education achieved the highest scores (*p* = 0.003 for N-QOL; *p* = 0.049 for Sleep/Energy; *p* < 0.001 for Bother/Concern) (Table 14).

Significant differences were also observed between participants with OAB-V8 scores < 8 and those with scores ≥ 8 across all three N-QOL scales (*p* < 0.0001 for each). There were no statistically significant differences associated with diabetes or the use of diuretic medications.

For the Sleep scale, scores declined significantly as the number of nocturia episodes increased, from 88.0 ± 12.7 among those waking once to 42.1 ± 20.9 among those waking five or more times (*p* < 0.0001). The Spearman correlation coefficient confirmed this negative relationship (r = −0.63, *p* < 0.0001). Women generally scored lower than men (60.2 ± 25.0 vs. 75.5 ± 20.9), although the difference approached but did not reach statistical significance (*p* = 0.057). Stratifying men according to the presence of BPH revealed a significant difference across three categories (men without BPH: 80.6 ± 18.3; men with BPH: 68.1 ± 22.3; women: 60.2 ± 25.0; *p* = 0.004). The lowest scores were observed among participants aged ≥ 80 years (60.4 ± 23.2), followed by those younger than 60 (68.4 ± 23.9), a group in which women outnumbered men (7 vs. 5). The highest scores corresponded to individuals aged 60–69 and 70–79 (79.2 ± 22.5 and 75.7 ± 17.2, respectively). Participants scoring ≥ 8 on the OAB-V8 scale showed lower performance on the Sleep scale (64.8 ± 22.0 vs. 89.3 ± 8.9, *p* < 0.0001). The presence of anxiety/depression was also associated with reduced scores (69.5 ± 23.3 vs. 80.3 ± 17.7). No statistically significant associations were observed with other demographic or clinical variables.

For the EQ-5D index, results were broadly similar to those of the Sleep scale. The difference according to nocturia frequency was not statistically significant under the Kruskal–Wallis test (*p* = 0.142), although Spearman correlation indicated a weak but significant negative association (r = −0.26, *p* = 0.013). This discrepancy reflects the aims of the two tests: the Kruskal–Wallis test assesses differences in medians across groups, whereas the Spearman coefficient evaluates monotonic relationships across the entire distribution. Women scored lower than men overall, and this difference increased when men were stratified by BPH status (men without BPH: 0.89 ± 0.19; men with BPH: 0.82 ± 0.24; women: 0.60 ± 0.24; *p* = 0.001). Participants aged ≥ 80 years had the lowest EQ-5D scores (0.65 ± 0.33), followed by those <60 years (0.72 ± 0.23), whereas individuals in the 60–69 and 70–79 age groups had the highest scores (0.87 ± 0.20 and 0.90 ± 0.15, respectively). Participants with OAB-V8 scores ≥ 8 demonstrated lower EQ-5D values (0.78 ± 0.25 vs. 0.92 ± 0.15; *p* = 0.011). No other demographic or clinical characteristics showed statistically significant associations with this index.

For the EQ-VAS scale, the number of nocturia episodes showed a clear effect, with scores declining from 78.1 ± 8.0 among individuals who woke once to 53.4 ± 12.7 among those who woke five or more times (*p* = 0.003). A significant negative correlation was also noted (r = −0.38, *p* = 0.0003). No statistically significant differences were found between men and women (*p* = 0.254) or with regard to BPH (*p* = 0.061). EQ-VAS declined with increasing age, from 74.7 ± 12.6 in individuals younger than 60 years to 54.6 ± 16.6 in those ≥ 80 years (*p* = 0.0008), with a corresponding correlation coefficient of r = −0.42 (*p* < 0.0001). Employment status was also associated with score differences: employed participants scored higher than unemployed individuals (76.1 ± 7.8 vs. 64.8 ± 14.4; *p* = 0.001). No other statistically significant associations were observed.

The Spearman correlation coefficients between the examined scales were statistically significant in all comparisons (*p* < 0.006). The strongest associations were observed among the Sleep/Energy, Bother/Concern, N-QOL, OAB-V8, and Athens Insomnia Scale scores, all of which demonstrated absolute correlation values greater than 0.69. In contrast, the EQ-5D and EQ-VAS scales showed weaker correlations with the other instruments (ranging from 0.33 to 0.56), and the weakest correlation was observed between EQ-5D and EQ-VAS themselves (r = 0.29) (Table 15).

At the conclusion of the study, an exploratory analysis was conducted to determine whether nocturia and its associated parameters were related to anxiety or depressive symptoms. To this end, the final item of the last questionnaire was used, capturing the respondent’s subjective experience of anxiety and distress at the time of assessment. The findings of this analysis are presented in Table 16.

With regard to symptoms of anxiety and depression, a borderline statistically significant difference was observed between men and women (57.7% vs. 90.9%, respectively; *p* = 0.046). However, this difference became borderline non-significant when men were stratified according to the presence or absence of benign prostatic hyperplasia (BPH). No statistically significant associations were found with respect to age, marital status, educational level, employment status, nocturia frequency, diabetes, or diuretic use.

Regression analyses were performed for all outcome measures, and in the case of the N-QOL instrument, separate models were constructed for its two subscales. The analysis indicated that increasing frequency of nocturnal awakenings was associated with a reduction in overall N-QOL score (regression coefficient: −6.7, 95% CI: −10.4 to −3.1). Furthermore, individuals scoring ≥8 on the OAB-V8 scale demonstrated poorer N-QOL performance (regression coefficient: −17.3, 95% CI: −26.7 to −8.0) (Table 17).

For the Sleep/Energy subscale, regression analysis demonstrated that increasing nocturia frequency was associated with a decline in performance (regression coefficient: −4.6, 95% CI: −6.8 to −2.4). Individuals with OAB-V8 scores ≥ 8 also had significantly lower Sleep/Energy scores (regression coefficient: −8.6, 95% CI: −14.4 to −2.9). In addition, women exhibited poorer performance compared with men without BPH (regression coefficient: −9.6, 95% CI: −16.7 to −2.5) (Table 18).

A similar pattern was observed for the Bother/Concern subscale. An increase in nocturia frequency was associated with a reduction in score (regression coefficient: −2.2, 95% CI: −4.0 to −0.3). Individuals with OAB-V8 scores ≥ 8 also demonstrated poorer performance on this subscale (regression coefficient: −8.7, 95% CI: −13.6 to −3.8) (Table 19). In contrast, participants with higher education exhibited better outcomes on the Bother/Concern scale (regression coefficient: 7.4, 95% CI: 0.6 to 14.2) (Table 18 and Table 19).

Regression analysis demonstrated that increasing nocturia frequency was associated with a decline in Athens Insomnia Scale scores (regression coefficient: −10.3, 95% CI: −13.8 to −6.9). Women also exhibited lower scores compared with men (regression coefficient: −13.5, 95% CI: −24.3 to −2.7). The presence of anxiety symptoms, included as an independent variable, was associated with reduced performance on the scale (regression coefficient: −7.0, 95% CI: −13.9 to −0.2). Additionally, a higher OAB-V8 score (≥8) was linked to a borderline statistically non-significant decline in Athens Insomnia Scale performance (regression coefficient: −8.5, 95% CI: −17.1 to 0.1) (Table 20).

In conclusion, regression analysis of health status demonstrated that increasing nocturia frequency was associated with a reduction in EQ-5D scores, although this effect was only borderline statistically significant (regression coefficient: −0.05, 95% CI: −0.094 to −0.0001). Women also exhibited lower EQ-5D scores compared with men (regression coefficient: −0.28, 95% CI: −0.423 to −0.133). A 10-year increase in age did not reach statistical significance at the 5% level (regression coefficient: −0.073, 95% CI: −0.122 to 0.005; *p* = 0.073) (Table 21).

Regression analysis of the EQ-VAS scale demonstrated that increasing nocturia frequency was associated with a reduction in self-reported health status (regression coefficient: −2.9, 95% CI: −5.4 to −0.3). A 10-year increase in age was also linked to lower EQ-VAS scores (regression coefficient: −5.4, 95% CI: −8.8 to −2.0). In contrast, being married was associated with higher perceived health (regression coefficient: 14.3, 95% CI: 5.6 to 23.1). Additionally, participants with OAB-V8 scores ≥ 8 tended to perform worse on the EQ-VAS scale (regression coefficient: −6.5, 95% CI: −12.5 to 0.4). Diabetes was also included in the model due to its known multifactorial effects on overall health status (Table 22). These findings provide a granular perspective on the components of daily functioning most affected by nocturia, which would not be evident from total scores alone. Given the absence of standardized Greek tools for nocturia evaluation, the item-level analysis provides valuable clinical information that may assist practitioners in understanding patient perceptions and priorities.

## 6. Discussion

Nocturia is a common lower urinary tract symptom that adversely affects sleep and quality of life, particularly among older adults. Despite its clinical importance, relatively few studies have focused specifically on its impact on patient-reported outcomes. The present study examined the burden of nocturia and its association with sleep quality and overall well-being. We observed a clear dose–response relationship between the frequency of nocturnal voiding and deterioration in patient-reported measures. Higher nocturia frequency was associated with poorer nocturia-specific quality of life, greater sleep disturbance, and lower perceived health status. These findings are consistent with previous research identifying nocturia as one of the most disruptive lower urinary tract symptoms, strongly linked to impaired daily functioning and reduced subjective well-being [1,2,3].

Daily fluid intake was not significantly associated with nocturia severity or any related clinical scales and was therefore excluded from the regression analyses. This finding may reflect seasonal variation, as data were collected during the winter months, when fluid consumption is typically lower. Similarly, neither diuretic use nor diabetes demonstrated a significant association with the examined outcomes. This is noteworthy given the well-established link between poorly controlled type II diabetes and nocturia [55,56,57].

In the international literature, Bosch et al. examined the causes of nocturia in males aged 60–80 years and found that multiple comorbidities—including hypertension, diabetes, heart failure, and neurological disorders—did not significantly differ in frequency between patients with and without nocturia [58]. These findings align with the current study, suggesting that nocturia cannot be explained solely by underlying systemic disease.

Sleep disruption appears to be a key mechanism linking nocturia to broader functional impairment. Fragmented sleep is associated with reduced REM sleep, diminished deep sleep stages, and prolonged sleep latency after reawakening. Previous studies have shown that nocturia contributes to sleep fragmentation in up to 75% of older adults, resulting in daytime somnolence, mood instability, and an increased risk of falls [6,20,21]. Our findings support this evidence, as participants with more frequent nocturnal awakenings consistently reported poorer sleep quality.

These findings carry important clinical implications. Given the strong association between nocturia frequency and impaired sleep quality, routine assessment of nocturia should extend beyond quantifying voiding episodes to include systematic evaluation of sleep disturbance and daytime functioning. Clinicians should actively inquire about sleep fragmentation, fatigue, and reduced productivity when assessing patients with nocturia, as these factors may substantially influence perceived disease burden. Incorporating validated instruments, such as sleep quality scales or targeted screening questions, may facilitate a more comprehensive evaluation. Furthermore, recognizing nocturia as a contributor to sleep disruption underscores the importance of multidisciplinary management approaches, including behavioral modification, optimization of comorbid conditions, and when appropriate, referral for sleep evaluation. Addressing nocturia in a structured and patient-centered manner may therefore improve not only urinary symptoms but also overall well-being and functional capacity.

From a practical clinical perspective, the results of this study may also help guide patient evaluation in routine practice. Individuals reporting three or more nocturnal voids per night may represent a subgroup at higher risk for clinically meaningful impairment in sleep quality and daily functioning. In such cases, the use of brief validated instruments—such as the Nocturia Quality of Life (N-QOL) questionnaire or the Athens Insomnia Scale (AIS)—may help clinicians identify patients whose symptoms extend beyond urinary inconvenience and significantly affect sleep and well-being. Early identification of this subgroup may facilitate timely intervention, including behavioral modification strategies, pharmacological treatment such as desmopressin in appropriately selected patients, or referral for sleep evaluation when sleep fragmentation appears disproportionate to urinary findings. Incorporating structured symptom assessment into routine urological consultations may therefore improve individualized management and help prioritize patients who could benefit from targeted therapeutic approaches.

The present findings demonstrate a clear dose–response relationship between nocturia frequency and all evaluated outcomes. As the number of nighttime voids increased, sleep quality, quality of life, and perceived general health progressively declined. Regression analysis showed that each additional nocturia episode was associated with a 6.7-point reduction in overall N-QOL score (95% CI: −10.4 to −3.1). Participants reporting a single nocturnal void had a mean score of 84.4 ± 13.4, whereas those reporting four episodes demonstrated a markedly lower score of 56.0 ± 19.7, reflecting substantial impairment in daily functioning. These results are consistent with large epidemiological studies indicating that two or more nightly awakenings are generally perceived as clinically disruptive [1,59,60]. In such studies, patients frequently report reduced vitality, daytime fatigue, and limitations in social and occupational functioning. Our finding that each additional nocturnal void corresponds to a measurable decline in quality of life further underscores the clinical relevance of nocturia, even in the absence of other lower urinary tract symptoms.

Comparable patterns were observed for both the EQ-5D and EQ-VAS measures. However, for EQ-VAS, nocturia frequency was not the strongest predictor of lower perceived health; age and marital status demonstrated more pronounced associations. This may reflect the subjective nature of the EQ-VAS, which captures overall perceived health rather than nocturia-specific functional impairment. Older age is often associated with a higher burden of comorbidities, potentially influencing global health perception. Although systematic reviews identify age as a major predisposing factor for nocturia—affecting sleep and quality of life in both sexes through hormonal changes and lower urinary tract aging [61]—this association was not strongly supported in the present study. In univariate analyses, age differences reached only borderline significance, with poorer outcomes observed mainly in individuals over 80 years. In multivariate models, age was generally not an independent predictor, possibly reflecting limited statistical power due to the modest sample size.

Emerging evidence also suggests that the developmental trajectory of nocturnal urinary symptoms may begin much earlier in life and extend into adulthood. A study by Yazici et al. investigated the relationship between childhood nocturnal enuresis and nocturia in young adults, finding that although a direct association was not significant in a general university population, the frequency of nocturia tended to increase among those with a history of severe enuresis [62]. This observation aligns with broader evidence indicating that nocturnal enuresis in childhood and nocturia in later life share common etiological pathways, including impaired bladder maturation, sleep–wake regulation abnormalities, and functional bladder capacity issues [63,64]. Indeed, meta-analytic data suggest that a history of childhood nocturnal enuresis is associated with a subsequent increased risk of developing nocturia in adulthood [63]. Moreover, population-based analyses have reported that adults whose nocturnal enuresis resolved at an older age (e.g., ≥12 years) exhibit higher rates of nocturia and urgency compared with those without such a history, highlighting the potential long-term impact of early urinary symptoms on adult bladder function [64,65]. These findings underscore the developmental continuity of nocturnal urinary dysfunction and suggest that longitudinal life-course perspectives may enrich our understanding of nocturia beyond its associations with aging and comorbidity.

Furthermore, nocturia remains a clinically heterogeneous symptom. In many cases, it is unclear whether individuals awaken due to urinary urgency or void after awakening for unrelated reasons. The decline observed in EQ-5D and EQ-VAS scores likely reflects a broader perception of impaired daily functioning rather than isolated urinary symptoms. Importantly, these findings do not indicate formal psychiatric morbidity; rather, they suggest that recurrent sleep interruptions may lead to discomfort, reduced energy, and increased concern about overall health. Similar patterns have been reported in population-based studies, where individuals with chronic nocturia describe poorer subjective health despite comparable comorbidity profiles [22,23,24].

Gender differences in nocturia remain underrecognized in the literature. Although both sexes experience nocturnal voiding, the underlying etiologies often differ. In men, benign prostatic hyperplasia (BPH) is a well-established contributor, whereas in women, reproductive history, childbirth-related pelvic floor trauma, and hormonal fluctuations—particularly after menopause—may play important roles. Most epidemiological studies report a higher prevalence in men across age groups [61]. For example, Chartier-Kastler et al. described substantial sleep disruption among men with BPH, including increased insomnia symptoms [66]. In contrast, Choo et al. reported a higher prevalence among women aged 18–49 years, with a threefold increase in the 18–29 age group [67].

In the present study, the limited number of female participants precluded reliable subgroup analyses. Notably, women consistently scored lower than men across all scales. To better account for disease-specific burden, male participants were stratified according to the presence or absence of BPH. This approach is justified, as nocturia is a core symptom of BPH and is known to negatively affect quality of life. Sarikaya et al. reported reduced sleep duration and increased daytime somnolence among patients with BPH [68]. Despite the high proportion of participants reporting BPH in our cohort, regression analysis did not demonstrate sustained statistical significance. This finding may reflect the use of men without BPH as the reference category, residual confounding, or potential underreporting of symptoms—particularly among older men.

No other demographic variables demonstrated consistent associations with the evaluated scales. Interestingly, individuals with technical education showed lower N-QOL scores, whereas married participants reported better overall health status. These findings may reflect underlying social dynamics. In the Greek sociocultural context, individuals with substantial occupational and familial responsibilities may prioritize external obligations over personal health. Conversely, those living alone may demonstrate greater attentiveness to symptoms or perceive them as more burdensome.

With respect to overactive bladder (OAB), the OAB-V8 questionnaire was initially employed for exploratory purposes. Nevertheless, meaningful findings emerged, notably that 64% of respondents scored ≥ 8, strongly suggestive of OAB. This condition remains underrecognized by both the general public and healthcare providers. Its true prevalence is likely higher than reported, as it is often investigated only in the presence of pronounced urgency or urge incontinence. Increased awareness and routine screening may therefore be warranted. Regarding nocturia, our results further support its multidimensional impact on sleep and quality of life, with symptom severity closely linked to the frequency of nocturnal awakenings. The strong association between elevated OAB-V8 scores and poorer quality-of-life outcomes is consistent with shared pathophysiological mechanisms between detrusor overactivity and nocturnal voiding. Urgency and increased daytime frequency reflect impaired bladder storage capacity, predisposing individuals to awaken during the night. Previous studies have shown that untreated or unrecognized OAB contributes to both the prevalence and severity of nocturia [41,42]. In our cohort, approximately two-thirds of participants exceeded the screening threshold, underscoring the importance of earlier identification and systematic assessment.

Another notable observation is the limited literature directly examining the relationship between nocturia and anxiety or psychosomatic symptoms. In the present study, only the sleep scale demonstrated a meaningful correlation with anxiety, likely reflecting the well-established association between sleep disturbance and anxiety disorders. Most participants reported moderate levels of anxiety, while 38.2% reported no anxiety and only two individuals described severe distress. These findings suggest that, although nocturia may contribute to emotional discomfort through sleep disruption, it does not necessarily indicate clinically significant psychiatric morbidity in the majority of cases.

The pathophysiology of nocturia is multifactorial, which likely contributes to its insufficient recognition as an independent clinical condition. Underlying mechanisms may be urological or systemic and vary considerably between individuals. Inadequate assessment can therefore lead to suboptimal treatment and unfavorable outcomes. Nocturia alone has been associated with impairment in 14 of 15 dimensions of health-related quality-of-life scales, with deterioration proportional to the number of nocturnal episodes. Although the symptom affects adults of all ages, younger individuals may experience a disproportionate impact due to occupational and caregiving responsibilities.

Beyond descriptive associations, evidence from interventional and disease-modifying studies further supports the clinical significance of nocturia and related lower urinary tract symptoms. Improvements in underlying bladder dysfunction or inflammatory urinary conditions have been shown to translate into measurable gains in sleep quality and health-related quality of life. For example, pharmacological management of overactive bladder and bladder inflammation has been associated with significant reductions in nocturnal voiding frequency and parallel improvements in patient-reported outcomes [69,70,71]. In related inflammatory bladder conditions, symptom-targeted therapy has demonstrated improvements not only in urinary frequency but also in fatigue and sleep disturbance, highlighting the interconnected physiological and behavioral dimensions of nocturnal urinary symptoms [72]. These findings reinforce the concept that nocturia represents a modifiable clinical burden rather than an inevitable consequence of aging.

From a translational perspective, our findings underscore the value of incorporating structured nocturia screening into routine primary care and urology assessments. Simple targeted questions regarding nighttime voiding frequency, sleep fragmentation, and daytime fatigue may facilitate earlier identification of clinically meaningful nocturia. Early recognition, particularly in older adults and women whose symptoms may be underreported, may enable timely behavioral, pharmacological, or multidisciplinary interventions and improve overall functional outcomes. Accordingly, these results reflect a real-world outpatient urology population and should not be directly generalized to the broader community or population-level settings.

Given its heterogeneous etiologies and complex management, further targeted research—particularly within the Greek population—is needed to better define its burden and develop effective intervention strategies. Taken together, these findings underscore that nocturia is not merely a urological complaint but a multidimensional clinical burden. Its impact extends beyond direct sleep interruption to secondary consequences such as reduced vitality, decreased productivity, and persistent concern about health status. A patient-centered understanding of nocturia may therefore help refine treatment priorities and support more individualized management approaches.

## 7. Limitations

This study has several limitations. First, its cross-sectional design prevents the establishment of causal relationships between nocturia and the examined clinical, psychological, or quality-of-life measures. The relatively small sample size (*n* = 89) further limits the statistical power of subgroup analyses and may increase the likelihood of type II error, particularly in comparisons involving women and older age groups. This relatively small sample size may limit statistical power and the ability to detect more subtle associations. The modest sample size also restricts the inclusion of more complex multivariable models and interaction analyses. Therefore, the findings should be interpreted with caution and considered exploratory, warranting confirmation in larger cohorts. Future studies with larger and more diverse populations are needed to validate and extend these findings.

Additionally, the study population was drawn from a single outpatient urology clinic, which may not be representative of the wider Greek population. Although recruitment was conducted consecutively during routine clinical practice, the outpatient-based design inherently reflects individuals actively seeking medical care and therefore may not capture asymptomatic or community-dwelling individuals with nocturia. Consequently, the findings should not be interpreted as population-level prevalence estimates but rather as an assessment of symptom burden and associated outcomes within a real-world clinical setting.

The composition of the study population should also be considered when interpreting the external validity of the findings. The majority of participants were older male patients attending a urology outpatient clinic, and therefore the results are most directly applicable to similar clinical populations. Caution is warranted when extrapolating these findings to women, younger individuals, or community-dwelling populations who may present different etiological patterns of nocturia. Future investigations should include larger and more diverse samples, ideally through multi-centre recruitment and incorporating objective measures such as frequency–volume charts or voiding diaries to strengthen diagnostic accuracy and improve generalisability.

To evaluate the robustness of the regression findings in the context of the limited number of female participants, exploratory sensitivity analyses were also performed in the male subgroup. These analyses yielded results consistent with the primary models, supporting the stability of the observed associations between nocturia frequency and the evaluated outcomes.

The predominance of retired male patients and the underrepresentation of women, particularly those from rural areas, may introduce selection bias and restrict the generalizability of the findings. The absence of exclusion criteria may have allowed confounding factors such as concurrent medical conditions, psychological stress, or medication use to influence symptom severity, but this reflects the real-world clinical burden of nocturia in our patient population.

Furthermore, the assessment of nocturia relied on self-reported data without confirmation through objective measurements such as frequency–volume charts, voiding diaries, or polysomnography. This introduces a potential recall bias and limits the ability to discriminate between nocturnal awakenings caused by urinary urgency and those attributable to unrelated sleep disturbances. The reliance on a single-item measure to assess anxiety and emotional distress constitutes another limitation, as it cannot capture the full clinical spectrum of psychological conditions such as generalized anxiety disorder or depression. Future studies should incorporate a standardized 3-day frequency–volume chart/voiding diary to better distinguish nocturnal polyuria from reduced bladder capacity and to quantify nocturnal urine production more precisely. Objective sleep assessment (e.g., actigraphy, and polysomnography when clinically indicated) would further improve the characterization of sleep fragmentation and help clarify whether voiding is the cause or the consequence of nocturnal awakenings.

Although we did not apply targeted exclusion criteria (e.g., screening for untreated obstructive sleep apnea), future studies should consider structured assessment or exclusion of major sleep disorders and incorporate objective sleep measures to reduce confounding. Larger cohorts would also permit more advanced adjustment strategies, including propensity-based approaches where appropriate, to better disentangle the independent contribution of nocturia to sleep disturbance and quality-of-life outcomes.

Moreover, the use of translated and culturally adapted questionnaires, although validated, may still introduce nuances in interpretation that differ from their original versions. The absence of formally validated Greek questionnaires specifically designed to assess nocturia further complicates the evaluation, potentially affecting the precision of results.

The characteristics of the rural Greek population represent an important contextual factor, as individuals—particularly women—may be less likely to seek medical consultation for urological symptoms. The relatively small sample size (*n* = 89), together with the limited recruitment period and the operational constraints of a morning outpatient clinic, limits generalizability. The underrepresentation of women further restricts the ability to draw conclusions regarding the broader female population. Many women preferentially consult gynecologists for urinary complaints, and such cases may therefore remain undocumented in urology clinics. Cultural norms, embarrassment, and limited health literacy may also discourage rural women from seeking specialist care. In our sample, most female participants were younger, employed, and residing in urban areas. Finally, nocturia is often underrecognized as a clinically relevant condition and may not prompt medical consultation, thereby influencing the composition of the study population. Additional contextual factors may include limited access to specialist services, transportation constraints, competing caregiving responsibilities, and a tendency to normalize urinary symptoms as part of aging or post-partum life. Such barriers may delay presentation and contribute to underrepresentation of women in urology outpatient settings, particularly in rural regions.

In addition, the number of exploratory statistical comparisons performed in this study may increase the probability of type I error, and therefore borderline statistical findings should be interpreted with caution and confirmed in larger cohorts.

Moreover, it is highly important to state that the findings of this study should be interpreted in light of its clinic-based design, single-center setting, and use of consecutive non-probability sampling. The study population consisted predominantly of older male patients from a specific regional healthcare setting, which may limit the generalizability of the results to broader or more diverse populations.

Finally, the study was conducted during winter months, which may have influenced fluid intake patterns and nocturnal behavior, limiting the ecological validity of the findings. Longer observational periods and more diverse seasonal data would likely yield a more accurate representation of nocturia patterns and their impact on daily functioning. Future studies should consider multicenter recruitment to increase sample size and improve representativeness, with targeted inclusion of women and younger adults. In addition, longitudinal designs are warranted to clarify temporal relationships and potential causal pathways between nocturia, sleep disturbance, and health-related quality of life. Such approaches would also allow evaluation of symptom progression and treatment-related changes over time.

## 8. Future Directions

Future research should build upon these findings by examining nocturia in larger and more diverse populations using multicenter or population-based sampling. Longitudinal designs would allow for a clearer understanding of the causal relationship between nocturnal voiding, sleep disruption, and quality of life, and would help distinguish whether nocturia is a direct driver of psychological burden or a consequence of other age-related comorbidities. Incorporating objective measures of sleep, such as actigraphy or polysomnography, alongside validated patient-reported outcomes could provide a more nuanced picture of the physiological and behavioral mechanisms that link nocturia to functional impairment.

In addition, future studies should explore the contribution of modifiable factors—such as hydration habits, medication use, or management of overactive bladder symptoms—and the interaction between nocturia and systemic conditions like diabetes or cardiovascular disease. Interventional research aimed at reducing nocturia episodes, improving sleep continuity, or enhancing patient awareness may help identify effective treatment strategies that address both clinical symptoms and quality-of-life domains. Ultimately, more comprehensive approaches that integrate urological, sleep medicine, and psychosocial perspectives are needed to improve care for individuals affected by nocturia.

## 9. Conclusions

Nocturia is a common and multifactorial condition that significantly impairs sleep quality, daily functioning, and overall well-being. Our findings show a clear dose-dependent relationship between the number of nocturnal awakenings and deterioration in quality-of-life measures, with overactive bladder contributing notably to this burden. Despite its prevalence, nocturia is often underestimated by patients and clinicians, leading to delayed diagnosis and suboptimal management. Recognizing nocturia as more than a benign consequence of aging is essential, and a comprehensive clinical approach that incorporates urinary symptoms, comorbidities, sleep disturbances, and psychosocial factors is required. Within this urology outpatient population, increasing nocturia frequency was associated with impaired sleep, reduced vitality, and diminished quality of life. Rather than reflecting background demographic or comorbid characteristics, the burden of nocturia is primarily linked to the frequency of night-time awakenings. These findings highlight the clinical relevance of nocturia in similar real-world clinical settings, although extrapolation to the general population should be made with caution. Further research in larger and more diverse populations is needed to better characterize the condition and to guide targeted interventions aimed at improving patient outcomes.

## Figures and Tables

**Table 1:** Causes of Polyuria.

Type of Diabetes	Subtypes of Diabetes
Diabetes Mellitus	Insulin-dependent (Type I) Non-insulin-dependent (Type II)
Diabetes Insipidus	Central (Pituitary) Nephrogenic Gestational Primary Polydipsia

**Table 2:** Causes of Nocturnal Polyuria.

Impaired Secretion or Action of Antidiuretic Hormone (ADH) Primary (Idiopathic) Secondary (Excessive Evening Intake of Fluids, Caffeine, or Alcohol
Congestive heart failure
Autonomic nervous system dysfunction
Sleep apnea syndrome
Renal failure
Estrogen deficiency

**Table 3:** Causes that lead to urinary storage problems.

Reduced functional bladder capacity (e.g., large post-void residual urine)
Reduced bladder capacity at night
Overactive bladder Neurogenic (e.g., multiple sclerosis) Non-neurogenic
Increased bladder sensitivity
Bladder obstruction with significant post-void residual urine
Advanced age

**Table 4:** Possible sleep disorders that may cause nocturia.

Insomnia
Sleep apnea syndrome
Restless legs syndrome
Tired legs syndrome
Parasomnias
Sleep disorders associated with medical conditions (e.g., COPD, heart diseases, etc.)
Sleep disorders related to neurological conditions (e.g., Alzheimer’s disease, Parkinson’s disease, epileptic seizures, etc.)

**Table 5:** Distribution, Mean (M), and Standard Deviation (SD) of Age by Gender (where N: number of individuals).

Gender	Men	Women	Total
Age	N (%)	N (%)	N (%)
<60	5 (6.4)	7 (63.6)	12 (13.5)
60–69	31 (39.7)	2 (18.2)	33 (37.1)
70–79	29 (37.2)	1 (9.1)	30 (33.7)
80+	13 (16.7)	1 (9.1)	14 (15.7)
Total	78 (100.0)	11 (100.0)	89 (100.0)
M (SD)	70.6 ± 9.21	54.1 ± 15.6	68.6 ± 11.5
*p*-value	<0.0001		

**Table 6:** The Number of Individuals and the Frequency of Nighttime Urination.

Nocturia	Number of Individuals (%)
1	8 (9.0)
2	36 (40.5)
3	26 (29.2)
4	9 (10.1)
5+	10 (11.2)

**Table 7:** Distribution of Responses to the N-QOL Questionnaire.

Category	Never or Not at All	Rarely/Sometimes or to a Small Extent/a Moderate Extent	Every Time/Most of the Time or to a Great Extent/an Excessive Extent	MeanScore ± Standard Deviation *
Sleep/Energy				
The problem of reduced sleep at night	21.4%	50.5%	28.1%	1.57 ± 1.20
Need for sleep the next day	24.7%	58.4%	16.9%	1.35 ± 1.12
Low energy the next day	31.5%	51.6%	16.9%	1.26 ± 1.14
Less productive the next day	39.3%	46.1%	14.6%	1.17 ± 1.17
Difficulty concentrating the next day	39.3%	45.0%	15.7%	1.15 ± 1.17
Less engagement in favorite activities	46.1%	43.8%	10.1%	0.96 ± 1.11
Disturbance/Worry				
Disturbance from waking up to urinate	5.6%	64.1%	30.3%	1.78 ± 0.97
Concern about the worsening condition	20.2%	49.5%	30.3%	1.71 ± 1.19
Concern about the lack of effective treatment	21.4%	55.0%	23.6%	1.56 ± 1.12
Concern about having to wake up to urinate	34.8%	38.2%	27.0%	1.38 ± 1.31
Concern about disturbing others in the house	62.9%	29.2%	7.9%	0.66 ± 1.01
Caution regarding water consumption	57.2%	40.5%	2.3%	0.53 ± 0.71

* The scale ranges from 0 (never or not at all) to 4 (always or to an excessive extent).

**Table 8:** Distribution of Responses to the OAB-V8 Questionnaire.

How Much Have You Been Bothered by	Not at All or Slightly	A Little or Moderately	Very or Extremely	MeanScore ± Standard Deviation *
Waking up at night because you had to urinate	37.1%	30.3%	32.6%	2.55 ± 1.32
Nocturnal urination	37.0%	31.5%	31.5%	2.53 ± 1.35
Frequent urination during the day	48.3%	39.3%	12.4%	1.74 ± 1.49
Sudden urge to urinate with little or no warning	82.0%	14.6%	3.4%	0.70 ± 1.15
Unexpected loss of a small amount of urine	84.2%	12.4%	3.4%	0.58 ± 1.04
Urine loss is associated with a strong urge to urinate	82.0%	16.9%	1.1%	0.54 ± 1.00
Unpleasant urge to urinate	86.5%	10.1%	3.4%	0.48 ± 1.00
Uncontrollable urge to urinate	84.2%	13.5%	2.3%	0.47 ± 0.99

* The scale ranges from 0 (not at all) to 5 (extremely).

**Table 9:** Distribution of Responses to the Athens Insomnia Scale Questionnaire.

Question	No Problem	Minor Problem	Moderate Problem	Severe Problem	MeanScore ± Standard Deviation *
**Night time awakenings**	1.1%	48.3%	42.7%	7.9%	1.57 ± 0.66
**Sleep Quality**	39.3%	41.5%	16.9%	23%	0.82 ± 0.79
**Total sleep duration**	44.9%	32.6%	19.1%	3.4%	0.81 ± 0.86
**Sleeponset**	44.9%	36.0%	14.6%	4.5%	0.79 ± 0.86
**Final awakening relative to the desired time**	51.7%	25.8%	18.0%	4.5%	0.75 ± 0.91
**Well-being the next day**	52.8%	33.7%	13.5%	0.0%	0.61 ± 0.72
**Sleepiness the nex tday**	60.6%	27.0%	9.0%	3.4%	0.55 ± 0.80
**Functionality the next day**	65.2%	25.8%	9.0%	0.0%	0.44 ± 0.66

* The scale ranges from 0 (no problem) to 3 (severe problem).

**Table 10:** Distribution of Responses to the EQ-5D Questionnaire.

Category	No Problem	Some Problems	Severe Problems	MeanScore ± Standard Deviation *
Anxiety/Depression	38.2%	43.8%	18.0%	1.80 ± 0.73
Pain/Discomfort	82.0%	16.9%	1.1%	1.19 ± 0.42
Mobility	86.5%	13.5%	0.0%	1.13 ± 0.34
Daily Activities	87.6%	12.4%	0.0%	1.12 ± 0.33
Self-Care	91.0%	9.0%	0.0%	1.09 ± 0.29

* The scale ranges from 1 (no problem) to 3 (severe problem).

**Table 11:** Health Conditions Recorded and the Corresponding EQ5D Score (MVH York A1 Tariff) by Descending Mean EQ VAS Score.

Health Condition	N	%	EQ5D (MVH York A1 Tariff)	Mean EQ VAS Score
11123	1	1.1	0.391	80.0
11113	11	12.3	0.504	72.7
11112	30	33.7	0.930	71.4
11111	30	33.7	1.000	71.3
22112	1	1.1	0.733	65.0
11122	3	3.4	0.817	61.7
11211	1	1.1	0.946	60.0
21223	1	1.1	0.256	50.0
22221	2	2.3	0.636	50.0
21222	2	2.3	0.682	46.5
22223	2	2.3	0.144	46.5
21123	1	1.1	0.306	45.0
22222	2	2.3	0.570	42.5
11232	1	1.1	0.248	40.0
22121	1	1.1	0.686	35.0
Total	89	100.0	0.826 *	67.6

* Mean value.

**Table 12:** Effect of Controlled Parameters on the OAB-V8 Score.

Parameter	OAB < 8 N (%)	OAB 8+ N (%)	Total N (%)
Nocturia			
1	7 (87.5)	1 (12.5)	8 (100.0)
2	22 (61.1)	14 (38.9)	36 (100.0)
3	2 (7.7)	24 (92.3)	26 (100.0)
4	1 (11.1)	8 (88.9)	9 (100.0)
5+	0 (0.0)	10 (100.0)	10 (100.0)
*p*-value		<0.0001	
Age			
<60	6 (50.0)	6 (50.0)	12 (100.0)
60–69	14 (42.4)	19 (57.6)	33 (100.0)
70–79	10 (33.3)	20 (66.7)	30 (100.0)
80+	2 (14.3)	12 (85.7)	14 (100.0)
*p*-value		0.040	
Gender			
Men	29 (37.2)	49 (62.8)	78 (100.0)
Women	3 (27.3)	8 (72.7)	11 (100.0)
*p*-value		0.740	
Marital Status			
Single/Divorced	5 (55.6)	4 (44.4)	9 (100.0)
Married	27 (33.8)	53 (66.2)	80 (100.0)
*p*-value		0.274	
Education			
Primary	21 (33.3)	42 (66.7)	63 (100.0)
Technical	3 (17.7)	14 (82.3)	17 (100.0)
Higher	8 (88.9)	1 (11.1)	9 (100.0)
*p*-value		0.034	
Employed			
No	20 (29.9)	47 (70.1)	67 (100.0)
Yes	12 (54.5)	10 (45.5)	22 (100.0)
*p*-value		0.036	
BPH (Benign Prostatic Hyperplasia)			
Men without BPH	25 (54.4)	21 (45.3)	46 (100.0)
Men with BPH	4 (12.5)	28 (87.5)	32 (100.0)
Women	3 (27.3)	8 (72.7)	11 (100.0)
*p*-value		0.001	
Diabetes			
No	25 (35.7)	45 (64.3)	70 (100.0)
Yes	7 (36.8)	12 (63.2)	19 (100.0)
*p*-value		0.928	
Diuretic Use			
No	17 (53.1)	29 (50.9)	46 (100.0)
Yes	15 (46.9)	28 (49.1)	43 (100.0)
*p*-value		0.839	

**Table 13:** Effect of Controlled Parameters on N-QOL and Subcategories.

Parameter	N (%)	Sleep/Energy (Min 0, Max 50)	Bother/Concern (Min 0, Max 50)	N-QOL (Min 0, Max 100)
Nocturia				
1	8 (9.0)	43.0 ± 8.2	41.4 ± 7.7	84.4 ± 13.4
2	36 (40.5)	41.4 ± 7.7	38.5 ± 7.2	79.9 ± 13.2
3	26 (29.2)	30.8 ± 12.9	31.2 ± 12.1	62.0 ± 23.2
4	9 (10.1)	26.9 ± 13.7	29.2 ± 10.0	56.0 ± 19.7
5+	10 (11.2)	18.0 ± 12.2	24.8 ± 6.8	43.8 ± 17.1
*p*-value		<0.0001	0.0003	<0.0001
Age				
<60	12 (13.5)	32.6 ± 13.1	36.3 ± 11.0	68.9 ± 23.4
60–69	33 (37.1)	37.4 ± 11.9	33.5 ± 11.5	70.8 ± 22.0
70–79	30 (33.7)	35.9 ± 13.2	36.5 ± 7.8	72.4 ± 18.7
80+	14 (15.7)	26.2 ± 13.4	28.9 ± 11.2	55.1 ± 23.4
*p*-value		0.066	0.198	0.145
Gender				
Men	78 (87.6)	35.6 ± 12.7	34.4 ± 10.5	70.0 ± 21.4
Women	11 (12.4)	26.3 ± 13.9	32.4 ± 10.4	58.7 ± 23.0
*p*-value		0.034	0.469	0.123
Marital Status				
Single/Divorced	9 (10.1)	34.7 ± 15.0	36.6 ± 12.2	71.3 ± 26.9
Married	80 (89.9)	34.5 ± 13.0	33.9 ± 10.3	68.3 ± 21.4
*p*-value		0.785	0.386	0.504
Education				
Primary	63 (70.8)	34.6 ± 13.4	33.3 ± 9.7	67.9 ± 21.5
Technical	17 (19.1)	29.7 ± 12.6	30.8 ± 11.6	60.4 ± 22.3
Higher	9 (10.1)	42.8 ± 7.3	46.1 ± 2.4	88.9 ± 8.4
*p*-value		0.049	<0.001	0.003
Employed				
No	67 (75.3)	33.7 ± 13.6	33.8 ± 10.4	67.5 ± 22.1
Yes	22 (24.7)	36.7 ± 11.7	35.2 ± 10.8	72.0 ± 21.1
*p*-value		0.372	0.613	0.458
Daily Fluid Intake				
<15 glasses	80 (89.9)	33.9 ± 13.1	33.8 ± 10.6	67.7 ± 21.8
15+ glasses	9 (10.1)	39.8 ± 12.8	37.2 ± 9.0	77.1 ± 21.4
*p*-value		0.066	0.417	0.173
OAB				
<8	32 (36.0)	44.8 ± 5.1	42.5 ± 4.5	87.3 ± 7.0
8+	57 (64.0)	28.7 ± 12.7	29.4 ± 9.8	58.1 ± 20.2
*p*-value		<0.0001	<0.0001	<0.0001
BPH				
Men without BPH	46 (51.7)	38.8 ± 10.9	37.1 ± 9.7	75.9 ± 19.1
Men with BPH	32 (35.9)	31.1 ± 13.9	30.5 ± 10.4	61.6 ± 22.1
Women	11 (12.4)	26.3 ± 13.9	32.4 ± 10.4	58.7 ± 23.0
*p*-value		0.004	0.016	0.005
Diabetes				
No	70 (78.6)	34.5 ± 12.5	34.6 ± 10.1	69.1 ± 20.8
Yes	19 (21.4)	34.4 ± 15.6	32.3 ± 11.6	66.8 ± 25.9
*p*-value		0.763	0.514	0.908
Diuretic Use				
No	46 (51.7)	33.8 ± 14.0	34.6 ± 11.0	68.3 ± 23.1
Yes	43 (48.3)	35.2 ± 12.2	33.7 ± 9.8	68.9 ± 20.6
*p*-value		0.811	0.630	0.990
Total	89 (100.0)	34.5 ± 13.1	34.1 ± 10.4	68.6 ± 21.8

**Table 14:** Effect of controlled variables on sleep and EuroQol score.

Variable	N (%)	Sleep (Min 0, Max 100)	EQ-5D (Min 0, Max 10)	EQ-VAS (Min 0, Max 100)
Nocturia				
1	8 (9.0)	88.0 ± 12.7	0.90 ± 0.16	78.1 ± 8.0
2	36 (40.5)	85.6 ± 11.6	0.90 ± 0.16	70.4 ± 9.1
3	26 (29.2)	69.4 ± 21.3	0.80 ± 0.25	68.2 ± 15.7
4	9 (10.1)	59.7 ± 15.5	0.65 ± 0.34	60.9 ± 17.4
5+	10 (11.2)	42.1 ± 20.9	0.74 ± 0.24	53.4 ± 12.7
*p*-value		<0.0001	0.142	0.003
Age				
<60	12 (13.5)	68.4 ± 23.9	0.72 ± 0.23	74.7 ± 12.6
60–69	33 (37.1)	79.2 ± 22.5	0.87 ± 0.20	72.0 ± 11.6
70–79	30 (33.7)	75.7 ± 17.2	0.90 ± 0.15	65.9 ± 11.3
80+	14 (15.7)	60.4 ± 23.2	0.65 ± 0.33	54.6 ± 16.6
*p*-value		0.027	0.017	0.0008
Gender				
Men	78 (87.6)	75.5 ± 20.9	0.86 ± 0.21	67.1 ± 13.6
Women	11 (12.4)	60.2 ± 25.0	0.60 ± 0.24	71.1 ± 16.4
*p*-value		0.057	0.002	0.254
Marital Status				
Single/Divorced	9 (10.1)	70.8 ± 31.0	0.77 ± 0.27	65.9 ± 13.6
Married	80 (89.9)	73.9 ± 20.8	0.83 ± 0.23	67.8 ± 14.0
*p*-value		0.821	0.321	0.717
Education				
Primary	63 (70.8)	73.2 ± 22.8	0.85 ± 0.20	66.3 ± 13.3
Technical	17 (19.1)	69.6 ± 21.1	0.73 ± 0.32	68.3 ± 16.3
Higher	9 (10.1)	83.8 ± 12.9	0.86 ± 0.20	75.3 ± 12.4
*p*-value		0.253	0.572	0.114
Employment				
No	67 (75.3)	72.2 ± 22.0	0.83 ± 0.24	64.8 ± 14.4
Yes	22 (24.7)	77.8 ± 21.4	0.82 ± 0.22	76.1 ± 7.8
*p*-value		0.165	0.839	0.001
Daily Fluid Intake				
<15 cups	80 (89.9)	73.1 ± 22.5	0.82 ± 0.24	66.6 ± 14.0
15+	9 (10.1)	77.8 ± 15.5	0.86 ± 0.20	76.4 ± 9.9
*p*-value		0.848	0.821	0.073
OAB (OveractiveBladder)				
<8	32 (36.0)	89.3 ± 8.9	0.92 ± 0.15	75.8 ± 8.1
8+	57 (64.0)	64.8 ± 22.0	0.78 ± 0.25	63.0 ± 14.4
*p*-value		<0.0001	0.011	<0.0001
BPH (Benign Prostatic Hyperplasia)				
Men without BPH	46 (51.7)	80.6 ± 18.3	0.89 ± 0.19	70.2 ± 11.9
Men with BPH	32 (35.9)	68.1 ± 22.3	0.82 ± 0.24	62.7 ± 14.7
Women	11 (12.4)	60.2 ± 25.0	0.60 ± 0.24	71.1 ± 16.4
*p*-value		0.004	0.001	0.061
Diabetes				
No	70 (78.6)	73.6 ± 21.3	0.84 ± 0.20	68.8 ± 13.1
Yes	19 (21.4)	73.5 ± 24.4	0.77 ± 0.31	62.9 ± 16.2
*p*-value		0.786	0.946	0.103
Use of Diuretics				
No	46 (51.7)	71.2 ± 22.4	0.81 ± 0.23	69.0 ± 14.6
Yes	43 (48.3)	76.2 ± 21.2	0.85 ± 0.23	66.1 ± 13.1
*p*-value		0.309	0.295	0.357
Anxiety/Depression				
None	34 (38.2)	80.3 ± 17.7		68.7 ± 13.8
Moderate/Excessive	55 (61.8)	69.5 ± 23.3		66.9 ± 14.1
*p*-value		0.034		0.458
Total	89 (100.0)	73.6 ± 21.8	0.83 ± 0.23	67.6 ± 13.9

**Table 15:** Spearman’s Correlation Coefficients between Scales.

Variable	Sleep/Energy	Distress/Anxiety	N-QOL	OAB-V8	AIS	EQ-5D	EQ-VAS
Sleep/Energy	-						
Distress/Anxiety	0.7307	-					
N-QOL	0.9379	0.9142	-				
OAB-V8	−0.7084	−0.6413	−0.7385	-			
AIS	0.8270	0.7052	0.8283	−0.6963	-		
EQ-5D	0.5106	0.3353	0.4539	−0.3869	0.4684	-	
EQ-VAS	0.5616	0.5210	0.5843	−0.5410	0.5223	0.2924	-

**Table 16:** Effect of Research Parameters on Reported Anxiety.

Parameter	No Anxiety (N, %)	Moderate or Excessive Anxiety (N, %)	Total (N, %)
Age			
<60	2 (16.7)	10 (83.3)	12 (100.0)
60–69	11 (33.3)	22 (66.7)	33 (100.0)
70–79	15 (50.0)	15 (50.0)	30 (100.0)
80+	6 (42.9)	8 (57.1)	14 (100.0)
*p*-value			0.078
Gender			
Men	33 (42.3)	45 (57.7)	78 (100.0)
Women	1 (9.1)	10 (90.9)	11 (100.0)
*p*-value			0.046
Marital Status			
Single/Divorced	2 (22.2)	7 (77.8)	9 (100.0)
Married	32 (40.0)	48 (60.0)	80 (100.0)
*p*-value			0.473
Education			
Primary	25 (39.7)	38 (60.3)	63 (100.0)
Technical	6 (35.3)	11 (64.7)	17 (100.0)
Higher Education	3 (33.3)	6 (66.7)	9 (100.0)
*p*-value			0.900
Employment			
No	26 (38.8)	41 (61.2)	67 (100.0)
Yes	8 (36.4)	14 (63.6)	22 (100.0)
*p*-value			0.838
Nocturia			
1	3 (37.5)	5 (62.5)	8 (100.0)
2	14 (38.9)	22 (61.1)	36 (100.0)
3	11 (42.3)	15 (57.7)	26 (100.0)
4	3 (33.3)	6 (66.7)	9 (100.0)
5+	3 (30.0)	7 (70.0)	10 (100.0)
*p*-value			0.972
BPH			
Men without BPH	22 (47.8)	24 (52.2)	46 (100.0)
Men with BPH	11 (34.4)	21 (65.6)	32 (100.0)
Women	1 (9.1)	10 (90.9)	11 (100.0)
*p*-value			0.051
Diabetes			
No	26 (37.1)	44 (62.9)	70 (100.0)
Yes	8 (42.1)	11 (57.9)	19 (100.0)
*p*-value			0.693
Use of Diuretics			
No	14 (30.4)	32 (69.6)	46 (100.0)
Yes	20 (46.5)	23 (53.5)	43 (100.0)
*p*-value			0.119
Total	34 (38.2)	55 (61.8)	89 (100.0)

**Table 17:** Regression Analysis for N-QOL.

Variable	Regression Coefficient (β)	*p*-Value	95% Confidence Interval (CI)
Nocturia Episodes	−6.7	0.0004	−10.4 to −3.1
Age (per decade)	−2.8	0.263	−7.7 to 2.1
Men without BPH (reference category)	-	-	-
Men with BPH	−1.9	0.643	−10.0 to 6.2
Women	−9.8	0.095	−21.4 to 1.7
Education	8.7	0.188	−4.4 to 21.8
Employed	−4.3	0.427	−14.9 to 6.4
Married	4.2	0.525	−8.9 to 17.4
Use of Diuretics	2.6	0.477	−4.6 to 9.7
OAB 8+	−17.3	0.0004	−26.7 to −8.0

**Table 18:** Regression Analysis for Sleep/Energy.

Variable	Regression Coefficient (β)	*p*-Value	95% Confidence Interval (CI)
Nocturia Episodes	−4.6	0.0001	−6.8 to −2.4
Age (per decade)	−2.3	0.135	−5.3 to 0.7
Men without BPH (reference category)	-	-	-
Men with BPH	−0.9	0.733	−5.8 to 4.1
Women	−9.6	0.009	−16.7 to −2.5
Education	1.4	0.738	−6.7 to 9.4
Employed	−1.3	0.694	−7.8 to 5.2
Married	2.5	0.533	−5.5 to 10.6
Use of Diuretics	2.2	0.324	−2.2 to 6.6
OAB 8+	−8.6	0.004	−14.4 to −2.9

**Table 19:** Regression Analysis for Distress/Anxiety.

Variable	Regression Coefficient (β)	*p*-Value	95% Confidence Interval (CI)
Nocturia Episodes	−2.2	0.025	−4.0 to −0.3
Age (per decade)	−0.5	0.703	−3.1 to 2.1
Men without BPH (reference category)	-	-	-
Menwith BPH	−1.0	0.625	−5.2 to 3.2
Women	−0.3	0.933	−6.3 to 5.8
Education	7.4	0.034	0.6 to 14.2
Employed	−3.0	0.288	−8.5 to 2.6
Married	1.7	0.625	−5.2 to 8.5
Use of Diuretics	0.4	0.839	−3.3 to 4.1
OAB 8+	−8.7	0.0006	−13.6 to −3.8

**Table 20:** Regression Analysis for Athens Insomnia Scale.

Variable	Regression Coefficient (β)	*p*-Value	95% Confidence Interval (CI)
Nocturia Episodes	−10.3	<0.001	−13.8 to −6.9
Age (per decade)	−4.1	0.088	−8.9 to 0.6
Women	−13.5	0.015	−24.3 to −2.7
Education	1.8	0.777	−10.6 to 14.1
Employed	−0.7	0.886	−10.9 to 9.4
Married	6.3	0.317	−6.2 to 18.8
Use of Diuretics	5.5	0.110	−1.3 to 12.3
OAB 8+	−8.5	0.053	−17.1 to 0.1
Feeling of Anxiety/Depression	−7.0	0.045	−13.9 to −0.2

**Table 21:** Regression Analysis for EQ-5D.

Variable	Regression Coefficient (β)	*p*-Value	95% Confidence Interval (CI)
Nocturia Episodes	−0.05	0.049	−0.094 to −0.0001
Age (per decade)	−0.06	0.073	−0.122 to 0.005
Women	−0.28	<0.001	−0.423 to −0.133
Education	−0.04	0.289	−0.110 to 0.033
Employed	−0.03	0.626	−0.170 to 0.103
Married	0.04	0.651	−0.127 to 0.202
Use of Diuretics	0.02	0.695	−0.075 to 0.112
OAB 8+	−0.07	0.251	−0.179 to 0.047

**Table 22:** Regression Analysis for EQ-VAS.

Variable	Regression Coefficient (β)	*p*-Value	95% Confidence Interval (CI)
Nocturia Episodes	−2.9	0.026	−5.4 to −0.3
Age (per decade)	−5.4	0.002	−8.8 to −2.0
Women	−0.2	0.960	−7.9 to 7.5
Education	2.8	0.158	−1.1 to 6.7
Employed	3.8	0.315	−3.6 to 11.1
Married	14.3	0.002	5.6 to 23.1
Use of Diuretics	0.3	0.918	−4.8 to 5.4
OAB 8+	−6.5	0.037	−12.5 to −0.4
Diabetes	−3.5	0.260	−9.5 to 2.6

## Data Availability

Data is unavailable due to privacy or ethical restrictions.

## Supplementary Materials

The following supporting information can be downloaded at: https://www.mdpi.com/article/10.3390/jcm15072492/s1, QUESTIONNAIRE.
