# Supplementary material for: Nocturia Frequency and Its Association with Sleep Disturbance and Health-Related Quality of Life in a Urology Outpatient Population
Source: J Clin Med. 2026 Mar 24;15(7):2492. doi: 10.3390/jcm15072492 (PMC13073114; doi:10.3390/jcm15072492)
Supplement: Supplementary file 1 [file jcm-15-02492-s001.zip › jcm-4115335-supplementary.pdf]

Supplementary Materials

QUESTIONNAIRE

Full Name:

Age: **Gender:** **Dateofbirth:**

Marital Status: Single ☐ Married ☐ Divorced ☐

Number of Children: ☐☐

Education Level:

1. No education ☐
2. Primary education ☐
3. Technical School ☐
4. University ☐
5. Master's degree ☐
6. Other:………………...

Occupation:

1. Unemployed ☐
2. Retired ☐
3. Farmer ☐
4. Public Sector Employee ☐
5. Private Sector Employee ☐
6. Self-employed ☐
7. Other: ……………………………………… ☐

Health conditions/issues:

Medication:

Dailyfluidintake:

In the last month, how many times did you usually need to get up to urinate from the moment you went to bed until the moment you woke up in the morning?

☐None
☐Once
☐Twice
☐Three times
☐Four times
☐ Five or more times

In the last 15 days, the fact that I had to get up during the night to urinate:

1. **It made it difficult for me to concentrate the next day**

☐ Every day ☐ Most days ☐ Some days ☐ Rarely ☐ Never

2. **Made me feel generally low on energy the next day**

☐ Every day ☐ Most days ☐ Some days ☐ Rarely ☐ Never

3. **Made me feel the need to sleep during the day**

☐ Every day ☐ Most days ☐ Some days ☐ Rarely ☐ Never

4. Made me less productive the next day

☐ Every day ☐ Most days ☐ Some days ☐ Rarely ☐ Never

5. **Forced me to engage less in activities I enjoy**

☐ Every day ☐ Most times ☐ Sometimes ☐ Rarely ☐ Never

6. **Made me pay attention to when and how much I drink**

☐ Every day ☐ Most times ☐ Sometimes ☐ Rarely ☐ Never

7. **Caused me trouble sleeping enough at night**

☐ Every night ☐ Most nights ☐ Some nights ☐ Rarely ☐ Never

8. **It made me worry I was disturbing others at home because I had to get up at night to urinate**

☐ To an excessive degree ☐ To a large degree ☐ To a moderate degree ☐ To a small degree ☐ Not at all

9. **Made me worry about having to get up at night to urinate**

☐ Constantly ☐ Most of the time ☐ Sometimes ☐ Rarely ☐ Never

10. **Made me worry that this condition would worsen in the future**

☐ To an excessive degree ☐ To a large degree ☐ To a moderate degree ☐ To a small degree ☐ Not at all

11. **Made me worry that there is no effective treatment for this condition (having to get up at night to urinate)**

☐ To an excessive degree ☐ To a large degree ☐ To a moderate degree ☐ To a small degree ☐ Not at all

12. **Overall, how bothersome was it to have to get up at night to urinate during the last 15 days?**

☐ Not at all ☐ To a small degree ☐ To a moderate degree ☐ To a large degree ☐ To an excessive degree

How bothered have you been by:

1. Frequent urination during the day
   ☐ Not at all ☐ Slightly ☐ A little ☐ Quite a bit ☐ Very much ☐ Extremely

2. Unpleasant urge to urinate
   ☐ Not at all ☐ Slightly ☐ A little ☐ Quite a bit ☐ Very much ☐ Extremely

3. Sudden urge to urinate with little or no warning
   ☐ Not at all ☐ Slightly ☐ A little ☐ Quite a bit ☐ Very much ☐ Extremely

4. Unexpected loss of a small amount of urine
   ☐ Not at all ☐ Slightly ☐ A little ☐ Quite a bit ☐ Very much ☐ Extremely

5. Nighttime urination
   ☐ Not at all ☐ Slightly ☐ A little ☐ Quite a bit ☐ Very much ☐ Extremely

6. Waking up during the night because you had to urinate
☐ Not at all ☐ Slightly ☐ A little ☐ Quite a bit ☐ Very much ☐ Extremely

7. Uncontrollable urge to urinate
☐ Not at all ☐ Slightly ☐ A little ☐ Quite a bit ☐ Very much ☐ Extremely

8. Loss of urine associated with a strong urge to urinate
☐ Not at all ☐ Slightly ☐ A little ☐ Quite a bit ☐ Very much ☐ Extremely

At least 3 days a week on average in the last month, one or more of the following occurred:

1. **Sleep onset**
☐ Very fast ☐ Slightly delayed ☐ Delayed ☐ Very delayed or did not sleep at all

2. **Awakenings during the night**
☐ No problem ☐ Minor problem ☐ Moderate problem ☐ Severe problem or did not sleep at all

3. **Final awakening compared to the desired time**
☐ At the desired time ☐ Slightly earlier ☐ Quite earlier ☐ Much earlier or did not sleep at all

4. **Total sleep duration**
☐ Adequate ☐ Rather inadequate ☐ Inadequate ☐ Very inadequate or did not sleep at all

5. **Sleep quality**
☐ Satisfactory ☐ Moderate ☐ Unsatisfactory ☐ Poor

6. **Well-being the next day**
☐ Fully ☐ Slightly reduced ☐ Quite reduced ☐ Very reduced or absent

7. **Functionality the next day**
☐ Full ☐ Slightly reduced ☐ Quite reduced ☐ Very reduced or absent

8. **Sleepiness the next day**
☐ None ☐ Mild ☐ Moderate ☐ Severe

Which of the following statements best describes your current health condition?

1. **Mobility**

a) I havenoproblemswalking

b) I havesomeproblemswalking

c) I ambedridden

2. **Self-care**

a) I have no problems with self-care

b) I have some problems with washing and dressing

c) I am unable to wash or dress myself

3. **Daily Activities (e.g., work, studies, household chores, family or social activities)**

   a) I have no problems performing my usual activities

   b) I have some problems performing my usual activities

   c) I am unable to perform my usual activities

4. **Pain/Discomfort**

   a) I feel no pain or discomfort

   b) I feel moderate pain or discomfort

   c) I feel excessive pain or discomfort

5. **Anxiety/Depression**

   a) I feel no anxiety or depression

   b) I feel moderate anxiety or depression

   c) I feel excessive anxiety or depression

6. **Compared to my health condition over the past 12 months, my current condition is**

   a) Better

   b) The same

   c) Worse

To help you express how good or bad your health condition is, we have placed a scale (like a thermometer) where the best condition you can imagine is at 100 and the worst condition you can imagine is at 0.
We would like you to mark on the scale how good or bad your current health condition is, based on your own assessment, with a line pointing to the point on the scale that reflects how good or bad your current health condition is.

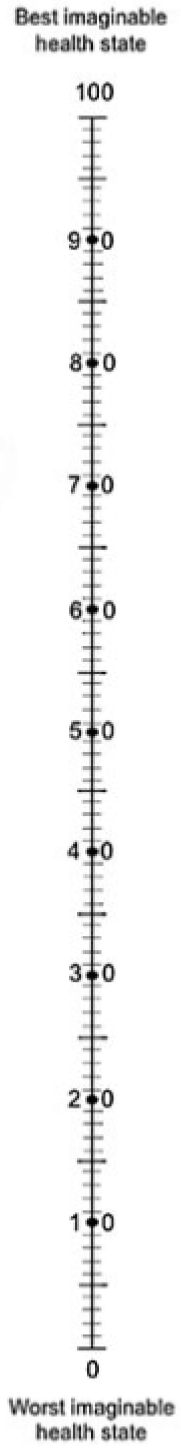

MARK EXACTLY THE POSITION/NUMBER THAT WAS INDICATED.

Your current health condition	
